# SAR.IoT: Secured Augmented Reality for IoT Devices Management

**DOI:** 10.3390/s21186001

**Published:** 2021-09-07

**Authors:** Daniel Fuentes, Luís Correia, Nuno Costa, Arsénio Reis, João Barroso, António Pereira

**Affiliations:** 1 Computer Science and Communication Research Centre, School of Technology and Management, Polytechnic Institute of Leiria, 2411-901 Leiria, Portugal; daniel.fuentes@ipleiria.pt (D.F.); luis.correia@ipleiria.pt (L.C.); nuno.costa@ipleiria.pt (N.C.); 2 INESC TEC, University of Trás-os-Montes e Alto Douro, Quinta de Prados, 5001-801 Vila Real, Portugal; ars@utad.pt (A.R.); jbarroso@utad.pt (J.B.); 3 INOV INESC Inovação, Institute of New Technologies, Leiria Office, Campus 2, Morro do Lena-Alto do Vieiro, Apartado 4163, 2411-901 Leiria, Portugal

**Keywords:** augmented reality, internet of things, IoT devices monitoring, IoT security, low-cost solution

## Abstract

Currently, solutions based on the Internet of Things (IoT) concept are increasingly being adopted in several fields, namely, industry, agriculture, and home automation. The costs associated with this type of equipment is reasonably small, as IoT devices usually do not have output peripherals to display information about their status (e.g., a screen or a printer), although they may have informative LEDs, which is sometimes insufficient. For most IoT devices, the price of a minimalist display, to output and display the device’s running status (i.e., what the device is doing), might cost much more than the actual IoT device. Occasionally, it might become necessary to visualize the IoT device output, making it necessary to find solutions to show the hardware output information in real time, without requiring extra equipment, only what the administrator usually has with them. In order to solve the above, a technological solution that allows for the visualization of IoT device information in actual time, using augmented reality and a simple smartphone, was developed and analyzed. In addition, the system created integrates a security layer, at the level of AR, to secure the shown data from unwanted eyes. The results of the tests carried out allowed us to validate the operation of the solution when accessing the information of the IoT devices, verify the operation of the security layer in AR, analyze the interaction between smartphones, the platform, and the devices, and check which AR markers are most optimized for this use case. This work results in a secure augmented reality solution, which can be used with a simple smartphone, to monitor/manage IoT devices in industrial, laboratory or research environments.

## 1. Introduction and Motivation

Currently, Internet of Things (IoT) solutions are becoming increasingly common in several areas (e.g., industry, agriculture, human location, and home automation) [1,2,3]. A key factor for their ease in adoption is the reasonable low cost of this type of equipment, which by not having relevant output peripherals such as an LCD displays can keep the costs low [4,5]. Considering that occasionally it is necessary to visualize the IoT device output or access real-time configurations, and that a simple LCD display device might cost much more than the IoT device itself, it becomes necessary to research solutions to visualize the IoT device data without the use of specific or additional equipment, only that already available to the administrator, namely a smartphone.

A solution with these characteristics can be used in different contexts such as configure information systems, support systems for the elderly to take medication, visualize the state of objects in a home, or even monitor industrial machines with IoT devices integrated. It is intended that the access to the information, of the various IoT devices, is done in a simple way, through a simple smartphone. Considering that the data to be accessed may be confidential, it is a main requirement to guarantee information security, guaranteeing that the data will only be available to those who have permission, and not to third parties. Considering that, in the scope of IoT systems, many of the equipment are ubiquitous with no information output interfaces, it is useful to use augmented reality to show this information in real-time near the device itself.

A possible scenario of this is a set of IoT devices scattered in a factory/farm that are controlling various sensors, namely temperature, pressure, and CO_2_, among others. For an administrator who needs to access real-time information from one of the IoT devices, assuming a universe of 1000 devices, it is necessary for the user to identify where the device is, see the identifier tag associated with it, access it and see the data that it is collecting, while assuming that the device has a web access interface or similar.

Using an augmented reality solution, where it is only necessary to point with an ordinary smartphone to visualize all the information on that device, and even access and configure it, makes the stated work in this article an interesting idea for this specific purpose. Although a solution with a dashboard that aggregates all the information from all devices is also useful and more common, using augmented reality enables the possibility of seeing the information of the device we are pointing to, in real-time, as if it had an output LCD display per example.

In this scope, the current work researches and presents a low-cost solution to monitor the status of IoT devices, in a secure way, using a simple smartphone and augmented reality.

## 2. Related Work

This section presents some works related to the theme of augmented reality (AR) associated with the Internet of Things. Some articles explained how augmented reality works and others have shown some solutions already implemented in the world of IoT.

Augmented reality combines information and virtual elements with real world imagery acquired through a camera. AR is becoming increasingly popular in common application for general public entertainment (e.g., gaming, video, and photo filters in social media mobile applications). In specific fields, there are other works, focused on marker detection, information security, platforms for interaction with devices, and IoT. Since this is one of the objectives of this document, works related to this theme will be addressed [6]. The implementation of the concept of augmented reality includes several types of technologies [7]: marker-based, marker less, projection-based, and overlay-based. Benefiting from a lower complexity in the interpretation of information, the type most widely implemented and used is augmented reality using a marker. In this approach, a camera and some type of marker is used, and the visual information is only shown when the marker is detected by a device using image or pattern recognition [8]. Ensuring that markers are detected with minimal latency time is a major challenge, and factors such as brightness and distance can affect marker recognition time [9,10].

Regarding previous research, one in particular has motivated a lot of interest—on which this solution was inspired—where the authors in [11], managed to use a smartphone and augmented reality to obtain the status of an IoT device, presenting some real examples. Additionally, a scalable AR framework called ARIoT was presented in [12], where the authors showed how a much friendlier environment makes use of AR to interact with the home IoT appliances. In [13], the focus was the benefits that augmented reality brings to public transport in smart cities and why it should always be used. Another interesting work is shown in [14], where augmented reality and a set of data information provided by IoT devices are used to locate the real position of various wireless transmitters. In the case of the platform presented in [15], it aims to make users aware of the energy consumption of the various electronic equipment in their home. For this purpose, the authors developed an interactive system that can display the energy consumption, measured by several IoT devices. This platform allows the user to visualize the energy consumption in real time and to interact with the device through AR. In the field of agriculture, there are also low-cost IoT solutions that provide real-time monitoring of crops [16], making the data visually available through AR. This work introduces the use of augmented reality as a support for IoT data visualization, also called AR-IoT. This concept superimposes the data collected from IoT devices directly to real-world objects and enhances the interaction with them. Regarding interior design, some applications that use AR technology have been developed, for example, in pre-sales, the customers can place and visualize furniture pieces inside their homes before purchasing them [17]. In assistive solutions, there are systems that use AR to assist people, for example, the authors of the work proposed in [18] developed a prototype that aimed to assist visually impaired people to read visual signs. The prototype consists of an augmented reality device, installed on top of the user’s head, which identifies real-world text (e.g., signs, room numbers, amongst others), highlights the location of the text, converts it into high-contrast letters through AR, and reads the content aloud through text-to-speech conversion. 

Most AR applications provide immersive virtual experiences by capturing information from the user’s environment and superimposing the virtual output to augment the user’s perception of the real world. The immersive interface and the user’s perception shift create serious safety and privacy concerns, mainly in situations where the AR information accuracy is crucial for the user (e.g., while driving a car). Because of this, it becomes essential to implement mechanisms to ensure that the information provided through AR is not affected by malicious applications or bugs [19]. 

The work proposed in the following sections of this paper was developed according to the concepts presented in [11,12,15,16] to create an information visualization system for IoT devices, in real-time, using augmented reality and adding a security layer to the AR. The work presented in [11] demonstrates how augmented reality can be used to expose information from IoT devices to the users, and in this case, using a simple smartphone to achieve that. The solutions shown in [12,15] confirm that the usage of AR to interact and monitor IoT devices is a valid option. In [16], although the focus is to use IoT devices and computer vision, it is not a solution designed to present information from IoT devices to the user, but to show information about something that the IoT devices are acquiring and processing from plantations. Even so, the assumptions exposed in this work and the information processing techniques are in accordance with what is necessary to the development of the work created in this article.

## 3. Conceptual Architecture

In this section, the conceptual architecture of the Secure Augmented Reality for Internet of Things (SAR.IoT) solution, oriented to the industry and research areas, is pressented. The main objective is to allow an augmented reality interaction between the user and the IoT devices, all through a web solution, and that guarantees the security of the information. The different modules are specified in detail below, namely the Client, Server and the IoT device.

**Figure 1:**
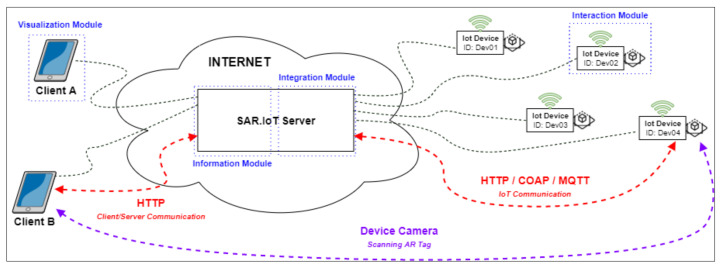
SAR.IoT conceptual architecture.

The SAR.IoT solution has a distributed and multi-agent architecture, as presented in Figure 1, which is mainly divided in three major roles (Client, Server, and IoT devices), in a total of four modules.

A distributed architecture [20] is composed of several modules that interact with each other in which each one is responsible for performing a specific task, and where the correct functioning of the entire system depends on the correct interaction of all the dispersed modules. A multi-tenant architecture allows having several customers/entities to interact with the system in general, while this interaction is carried out through the use of credentials that guarantee the privacy of the data. 

This proposed architecture was specified considering the communication architecture most widely used in the IoT universe, the Client/Server architecture [21], having been properly modified to incorporate all the requirements necessary for the smooth functioning of the solution. The architecture comprises four modules:

- The visualization module, acting in the client role, acquires and processes images to identify possible markers. In the case of a positive identification, it queries the information module for the data related to the identified marker;
- The information module, acting in the server role, stores the data related to the IoT devices and their associated AR markers. It replies to requests from the display module and assures the security of the information;
- The integration module, also acting in the server role, provides communication between the IoT devices and the information module; and
- The interaction module, acting in the IoT role, provides interaction between the integration module and the IoT device and is located on the device itself.

Each module has an agent, a software-based entity, which is responsible for performing various tasks and ensuring the efficient operation of the overall system.

### 3.1. Client

**Figure 2:**
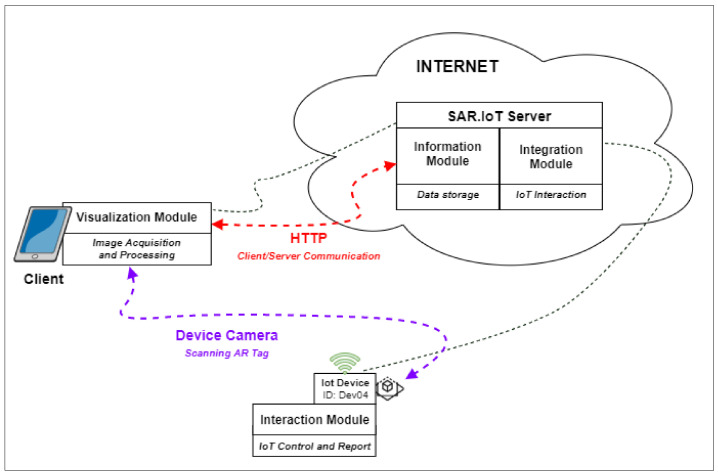
Client role.

The interconnection between the Client, Server, and IoT device can be seen in Figure 2. Note that although there is a direct interaction between the Client and IoT device, this only occurs for the AR marker reading associated with the device, all the communications were performed using the server.

**Figure 3:**
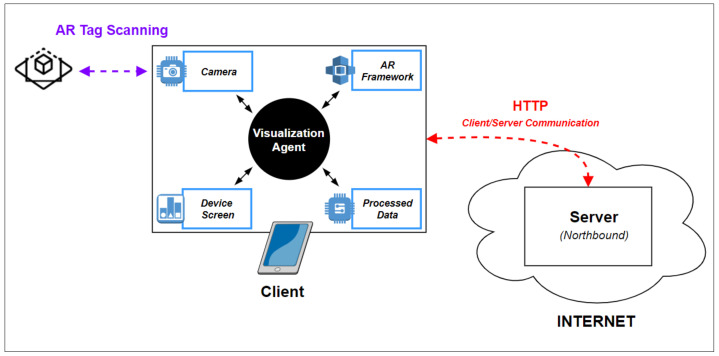
Visualization module architecture.

The client is a user device such as a smartphone and hosts the visualization module installed and its software agent. This agent acquires and analyzes images to extract AR markers using the resources available in the client device and the augmented reality framework implemented in the solution, as displayed in Figure 3, allowing the interaction using the smartphone screen.

After identifying the marker, the agent queries the information module present on the server via the server’s northbound interface to obtain the related data and to display it on the screen of the client device.

### 3.2. Server

**Figure 4:**
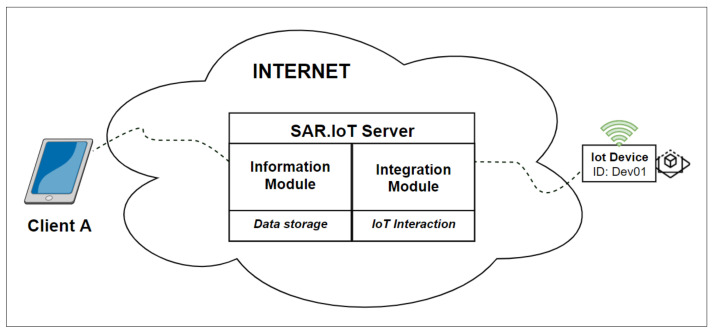
Server role.

Figure 4 shows part of the proposed architecture for the server, where it is possible to see the two modules within it, the information module, and the integration module, each performing their respective tasks, interacting with each other and with the other modules via northbound and southbound, respectively.

**Figure 5:**
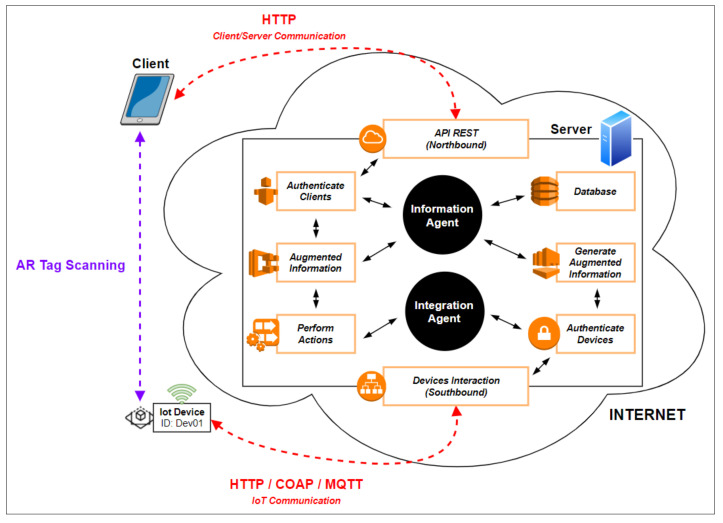
Visualization module and integration module architecture.

The server hosts the information agent and the integration agent, as shown in Figure 5. The server authenticates and replies to the requests from the clients and authenticates and receives information from the IoT devices. The information agent receives, processes, and replies to the requests made to the server. The integration agent receives information from the IoT devices and forwards it to the information agent for data generation and storing. This last agent also performs the actions on the IoT devices such as enabling output, etc.

The server stores the configuration information as well as all the information sent by the IoT devices. It manages the identification, authentication, and access of IoT devices and users, making the access to the data secure.

### 3.3. IoT Device

**Figure 6:**
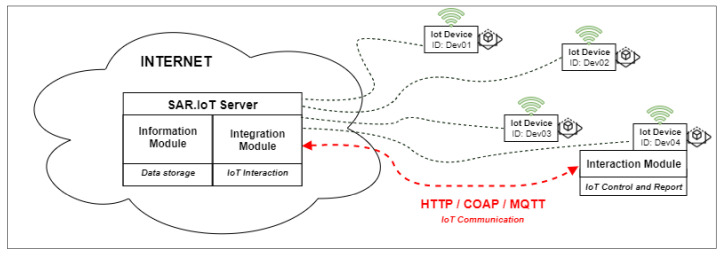
IoT device role.

In order to be able to interact with an IoT device, it is necessary to be able to communicate with it, either to obtain information about the equipment itself or to perform actions. For this to be possible, it is necessary to provide this IoT device with a software-based agent that returns the needed information and performs the desired actions. Figure 6 shows the zoom at this point in the general architecture, where it is possible to visualize the connection between the server’s integration module and the IoT devices of the interaction module.

**Figure 7:**
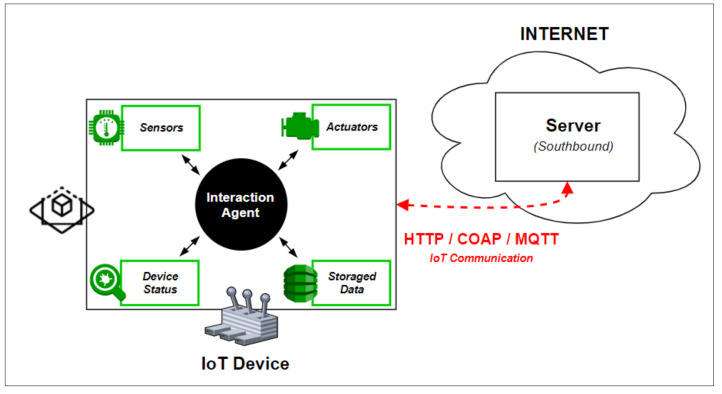
Interaction module architecture.

The agent present in the interaction module, shown in Figure 7, allows for the collection of all the information within the sensors associated with the IoT device, performing actions on the outputs, accessing information about the device itself, among others. The interaction module is responsible for communicating with the server through the integration module and sending/receiving all the data necessary for the system to function.

### 3.4. Communication

**Figure 8:**
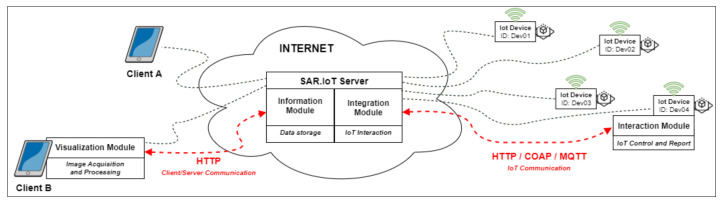
Communication architecture.

In Figure 8, part of the proposed architecture is presented, focused on the different communication protocols incorporated in it.

In the communication between the different modules in the architecture, and since it is an IoT environment, it makes sense to use communication protocols suitable for this purpose. In the previous figure, there are two different types of communication: northbound communication between clients and the server, accessing the information module; and southbound communication between the integration module on the server and the interaction module on IoT devices.

In the northbound communication, since this typically occurs in a web environment over the Internet, the communication protocol to be used will undoubtedly be HTTP (Hypertext Transfer Protocol)—in its secure version, HTTPS—to ensure data security. This is one of the most widely used protocols [21] for accessing online platforms and is widely used in the IoT environment for the same purpose.

In the southbound communication, since it is mostly communication between IoT devices and the server, several protocols focused on the IoT environment can be used:

- HTTP (Hypertext Transfer Protocol): The most used Client/Server communication protocol on the web, which is also widely used in the IoT world due to its simplicity and efficiency in the delivery of information;
- COAP (Constrained Application Protocol): A communication protocol designed for devices that have limited processing capabilities, very similar to HTTP, but uses much less data when sending messages; and
- MQTT (Message Queuing Telemetry Transport): One of the lightest communication protocols, uses the Publisher/Subscriber model to exchange messages and is widely used in scenarios where network connectivity is not ideal.

These are just some examples of some of the communication protocols most widely used by programmers that can be applied to this architecture. In southbound communication, the use of HTTPS is recommended, the secured version of HTTP. The protocols COAP and MQTT can also be used, but only with an implementation of the protocols that ensure the data security, namely Lithe [22] and SMQTT [23].

## 4. Prototype Implementation

This section presents the prototype developed to test and validate the proposed architecture, describing the analyzed frameworks, the used equipment, and the operation of the entire solution. For this project, a solution was developed, mostly focused on web technologies, called the Secured Augmented Reality for IoT, shortly named SAR.IoT.

### 4.1. Frameworks, SDKs, and Augmented Reality Libraries

Below is presented a review of the four most widely used and currently available SDKs. For this project, we selected the ARToolkit SDK, a choice justified at the end of the subsection.

#### 4.1.1. Vuforia

The Vuforia SDK [24] is one of the most popular augmented reality SDKs to develop AR solutions for Android, iOS, UWP, and Unity. It can recognize images, objects, and text. It uses simultaneous localization and mapping (SLAM) technology, which makes it possible for applications to recognize 3D scenes and objects. Regarding the licensing, Vuforia is free for development.

#### 4.1.2. Apple ARKit

The Apple ARKit framework [25] was introduced in iOS11 and allows for the creation of augmented reality applications for iPhone and iPad. It can recognize images, objects, and text. It also uses SLAM technology in conjunction with the device’s built-in sensors. Regarding the licensing, the platform is free, but it only works on Apple devices running iOS11+ and with A9, A10, and A11 processors.

#### 4.1.3. Google ARCore

Google’s ARCore SDK [26] was designed to support the creation of AR applications for Android 7.0+ devices. It can also recognize images, objects, and text. It also uses SLAM technology in conjunction with the device’s built-in sensors. Regarding the licensing, the platform is free, but only works on Android and iOS devices along with ARKit.

#### 4.1.4. ARToolkit

ARToolKit [27] is a free open-source library, from version 5.2 onward (GPLv3), which can be used to create cross-platform AR applications including Android, iOS, UWP, Unity, and Web solutions. It can recognize images, text, and NFT (Natural Feature Tracking) and was used in many previous works [28] with success. Since one of the defined goals is the use of a web platform, it was selected to implement the presented solution.

### 4.2. Equipment Used in Prototype

**Figure 9:**
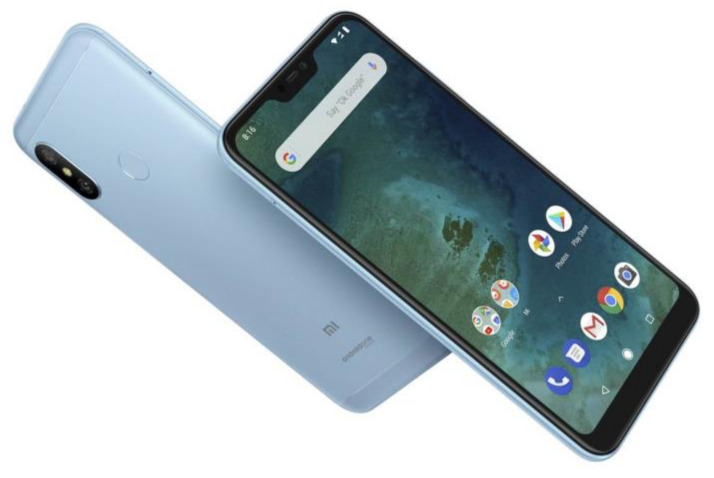
Smartphone Android Xiaomi A2 Lite.

The economic value is an important issue for the device to be used in this project, so we opted to use ordinary equipment, readily available to most users. For the client device (i.e., to capture images and process the augmented reality), we selected an Android [29] smartphone, displayed in Figure 9, priced around €150.

**Figure 10:**
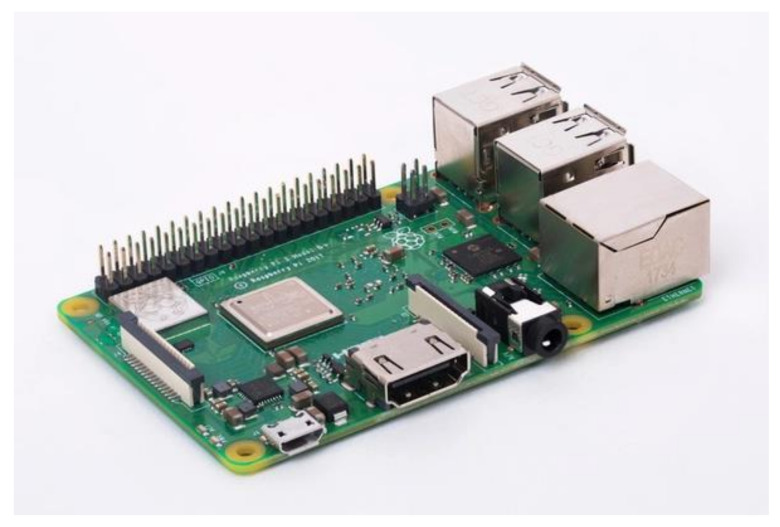
Raspberry Pi 3 B+.

For the server equipment (i.e., which receives and replies to requests, stores information from the IoT devices, and authenticates users and IoT devices), we selected a Raspberry Pi 3 B+ [30], displayed in Figure 10, priced at €50 including a 5 V 2.5 A power supply and a Micro SD card. The characteristics of this equipment, despite being an IoT device, are suitable for the intended purpose, since it includes a quad-core 1.4 GHz processor and 1 GB of RAM, which assures sufficient server performance.

### 4.3. Operation

To present augmented information in a device, it is necessary to analyze and process the image captured by the device itself, usually using a proper software library. In this case, we used the ARToolKit library, the Javascript version, JSARToolKit5 [31], to work in a web environment, allowing the system to be used by any device with an updated browser (e.g., smartphones, tablets, computers, etc.). To create the augmented information in Javascript, we used the Three.js library, commonly used for WebGL 3D development. Together with the ARToolKit, we. used the Threex.ARToolKit [32], which is also used by the AR.js library [33].

**Figure 11:**
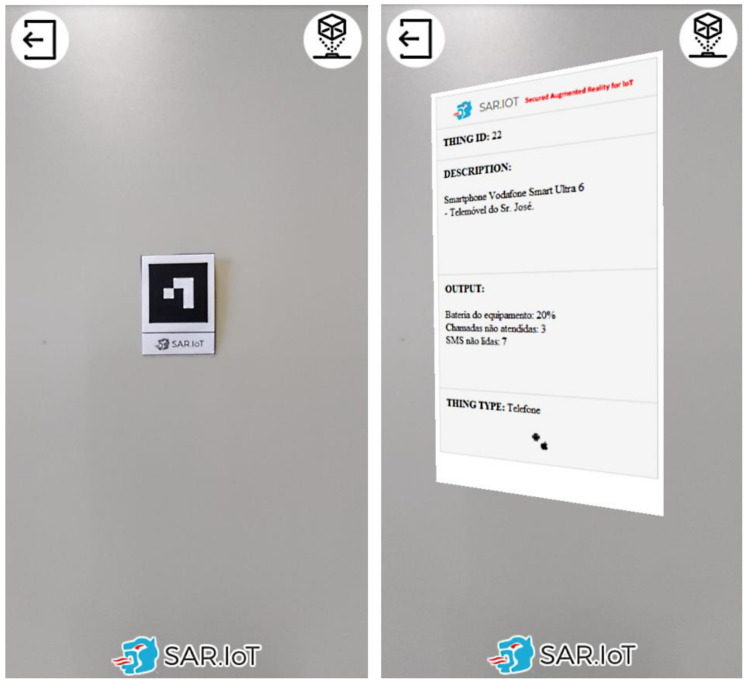
AR marker (**left**) being detected and processed (**right**).

For this solution, a web platform was developed using HTML, PHP, MySQL, and Javascript, and is accessible by the smartphone devices, though the HTTPS protocol (the secured version of HTTP). The client-side data processing is executed in Javascript on the smartphones and includes the AR marker detection and the augmented content rendering systems, as displayed in Figure 11.

**Figure 12:**
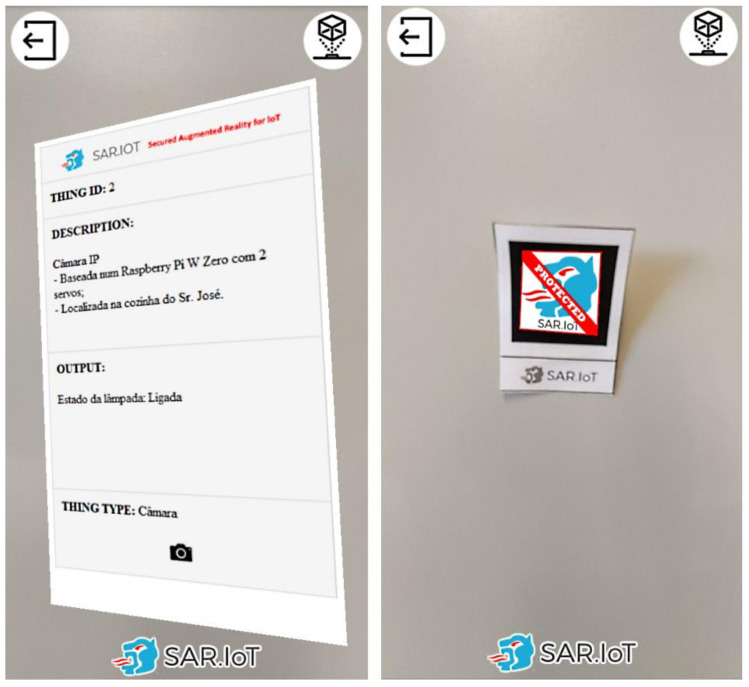
AR markers with (**left**) and without (**right**) access granted.

Each client can only see the information of the markers to which it has been granted access by the system administrator. Figure 12 presents an example of this security feature in operation. In the left side is displayed a marker with augmented content to which the user has access rights, and in the right side is displayed a marker to which the user does not have access rights, together with a access restriction notification.

By doing this, it is possible to have a secure system where only authorized persons can access the information of the devices. The advantage in this method is that the user is able, in real-time, to have a sense of what is happening with the devices, all of this without the need to read codes manually, and subsequently accessing a URL with the device information, in the case of using QR-Codes. It should also be noted that the ease of use of an augmented reality system, in an IoT scenario with these characteristics, is an asset for any multi-user implementation because there is no need to physically interact with the IoT devices.

**Figure 13:**
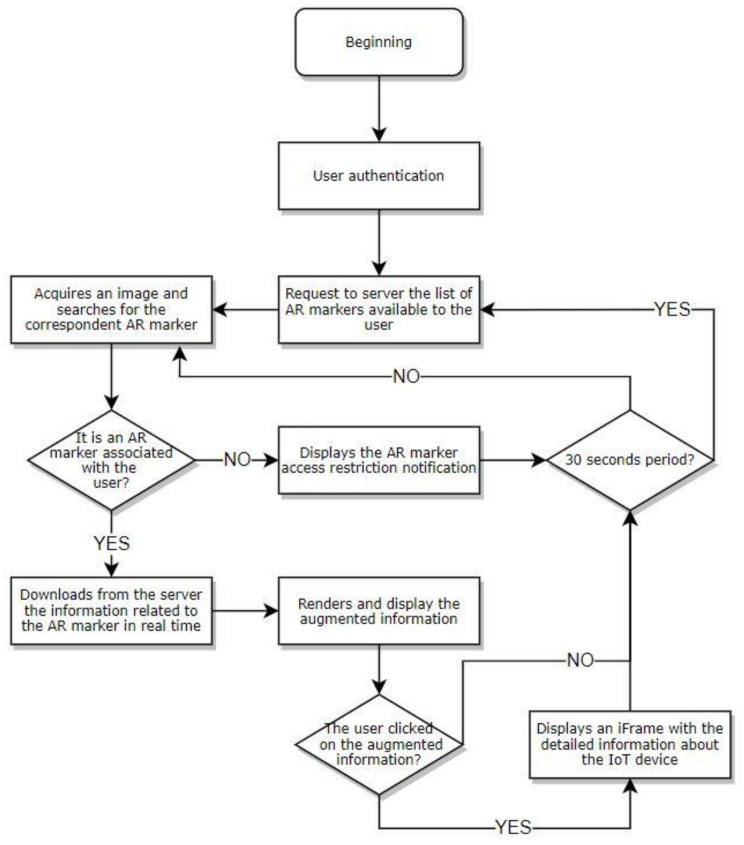
Process view of the client’s operation.

The diagram in Figure 13 presents a process view of the client operation, detailing the actions performed by each activity of the process.

That same operation occurs in the following order:

• The process begins when the client logs into the platform and the marker detection system is started;
• The client (client-side Javascript) sends a request to the server for the bookmarks associated with the current user;
The mobile device captures images and the client searches for AR markers in the images; and
• When a marker is detected, the system checks whether it can be displayed to the current user:
◦ If yes, the system downloads the information about the marker from the server and renders and displays it on the device’s screen (augmented reality). If the user clicks on the augmented information, an embedded webpage is displayed with the full information regarding the IoT device.
◦ If not, the system displays a marker access restriction notification.

**Figure 14:**
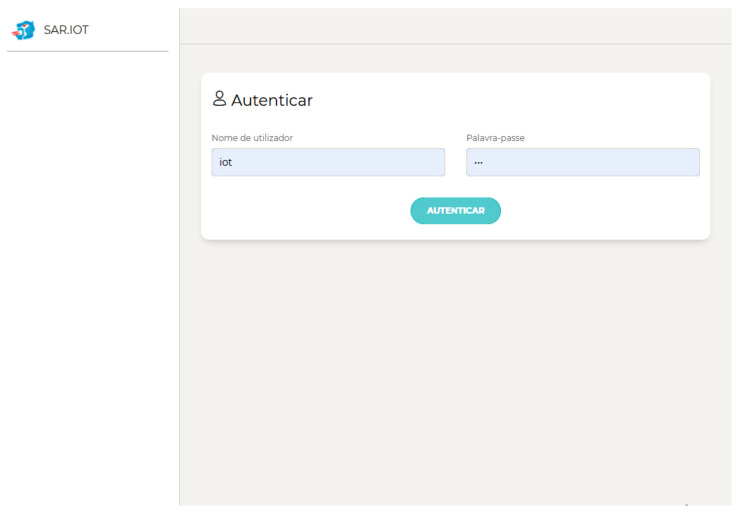
Authentication page.

The system refreshes the information from the markers and devices every 30 s and the authentication activity is mandatory, as shown in Figure 14. If the user has an administration profile, it is redirected to the platform management portal. Otherwise, the client process starts as previously described.

**Figure 15:**
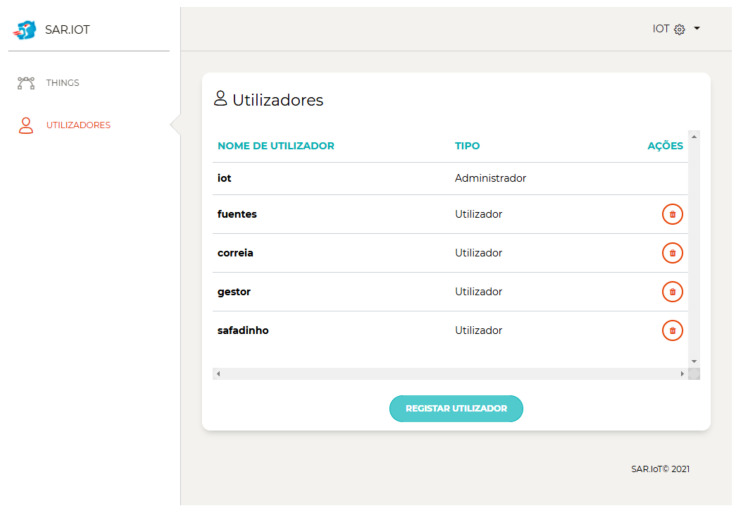
User listing.

The SAR.IoT platform includes the management features (i.e., user profiles, IoT devices, and bookmarks and associations). Figure 15 shows the users’ management page used for Create, Read, Update, and Delete (CRUD) operations, where there can exist normal users or administrators.

**Figure 16:**
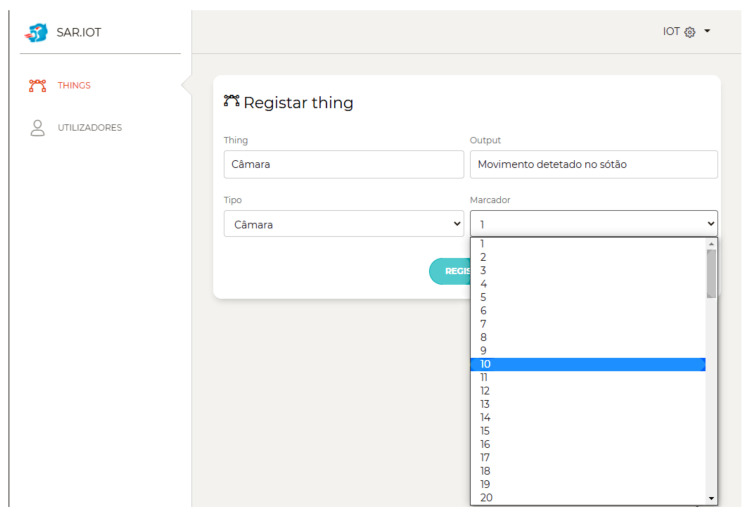
Thing parameters.

Figure 16 shows the IoT device management page with CRUD (Create, Read, Update, and Delete) operations. The IoT devices can specify the device types, the AR markers, and users that can interact with them.

**Figure 17:**
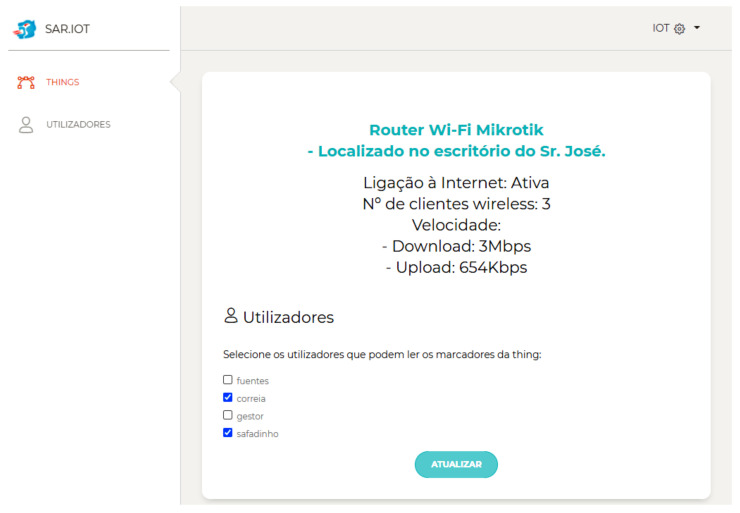
Association between an IoT device and users.

Figure 17 shows the page to manage the association between IoT devices and users for access purposes.

### 4.4. Visualization Modes

**Figure 18:**
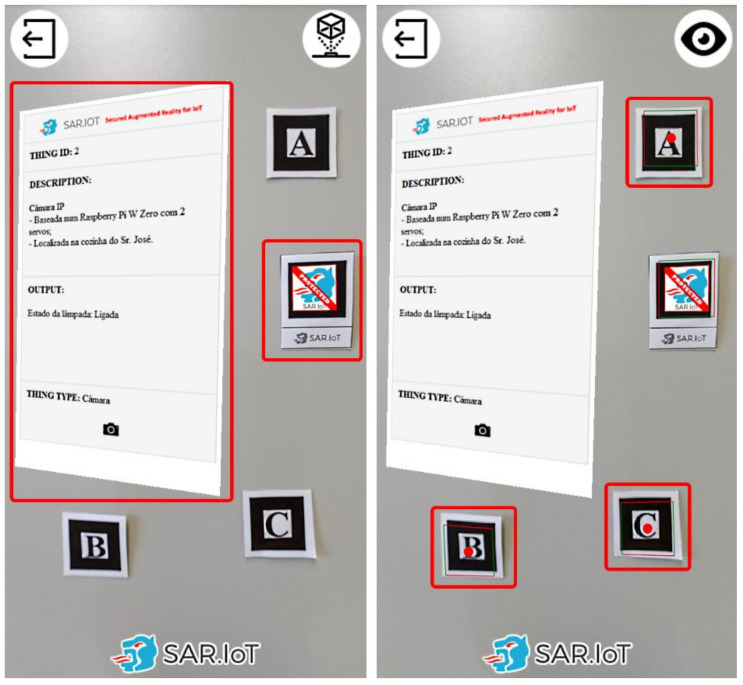
Normal mode (**left**) and debug mode (**right**).

In the visualization module, two visualization modes were implemented: a normal mode and a debug mode, as displayed in Figure 18. The normal mode presents the IoT device’s augmented information, while the debug mode adds graphical pins to signal the presence of augmented information that is not available or configured in the system. To switch between modes, the user can click the button on the upper right screen corner.

The debug mode is very useful in situations where we want to confirm whether the mobile device (smartphone) is able to recognize the AR marker or not. This is implemented because there may be situations in which the equipment does not present any information and may not be able to read the tags due to some defect on them. With this, it is possible to know if the system is working correctly or not, if it just cannot obtain the information from the server, or if there is another problem with the client module, namely reading the markers.

### 4.5. Interaction with Augmented Reality

**Figure 19:**
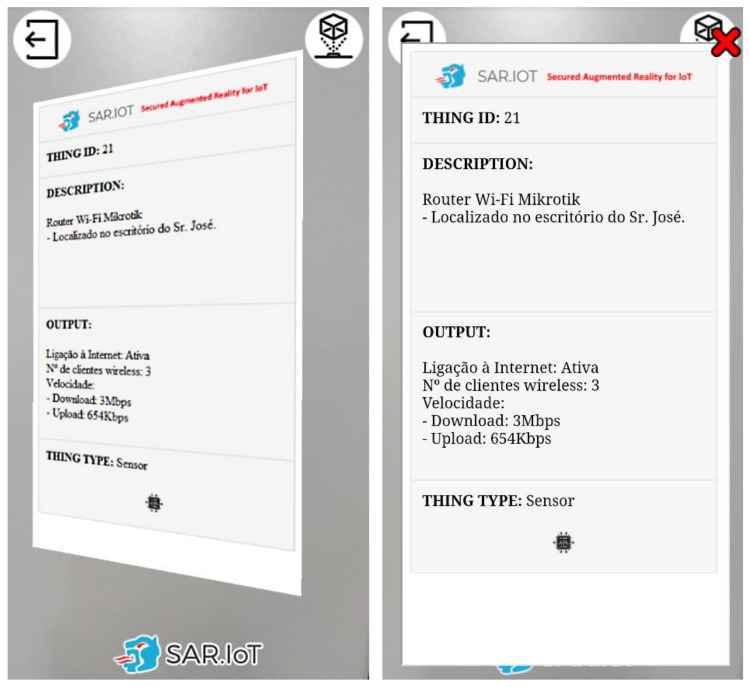
Processed AR marker (**left**) and the correspondent interaction dashboard (**right**).

When an AR marker augmented reality information in clicked/pressed on the smartphone’s screen, an embedded web page with content regarding the IoT device is produced, as displayed in Figure 19. The contents are updated in real time and can allow interaction with the IoT device.

This available information is acquired by the interaction module and sent to the server through the integration module. It was decided to implement this interaction system to allow the user to consult the information of the IoT devices more comfortably, without the need to be pointing to the marker. The idea in this approach is for the user to be able to verify in real-time the information of all the IoT devices that they can see, but if they want to interact or analyze the information in detail, when clicking on the information generated in AR, a new window appears with all the information that was in the AR information and other further details.

### 4.6. An Augmented Monitoring Tool

**Figure 20:**
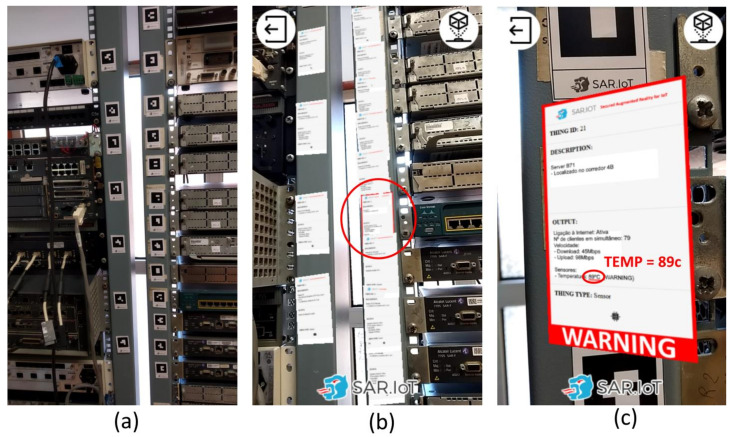
AR markers (**a**) being detected (**b**) and showing an anomaly in a device (**c**).

The great advantage of an augmented reality system incorporated in a smartphone, is its ability to easily allow the detection of equipment that is malfunctioning by simply pointing the smartphone’s camera to the IoT devices and checking if any of them have warnings. In the example shown in Figure 20, it is possible to observe a scenario where multiple IoT devices have an AR marker attached (a), when using the proposed system, one of the multiple AR information windows visible in the smartphone is drawing attention (b), that is, there is a problem with that specific device that needs to be checked, doing that by simply closing in the smartphone and seeing what is happening (c). When using a simple dashboard on a smartphone, it indicates that there is a device with problems, but the user must search for the physical equipment and, typically, for written tags with their identification until they find it. 

The main benefit of the given solution is that it transforms any smartphone in a real-time monitoring tool, for example, allowing better monitorization of all the IoT devices in an industry or a laboratory.

## 5. Tests and Optimizations

In this section, all the tests and optimizations performed are exposed. Various types of AR markers were tested and different degrees of confidence in the recognition system were analyzed. The performance of the solution was also analyzed, with multiple AR markers being shown in the screen at the same time. The security layer of the AR markers was also tested, namely, if the system could block access to the information of the markers to unauthorized users.

### 5.1. Types of AR Markers

**Figure 21:**
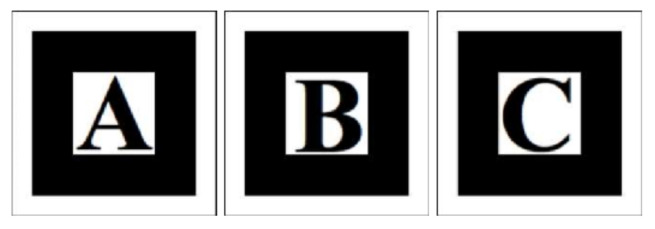
PATTERN markers.

During the development, we tested PATTERN, 2D BARCODE, and NFT (Natural Feature Tracking) markers, the AR markers used in the tests had a size of 2.5 cm × 2.5 cm, as the ones in Figure 21 that is showing some pattern markers, and the distance of the readings taken with the smartphone varied between 5 cm and 50 cm.

**Figure 22:**
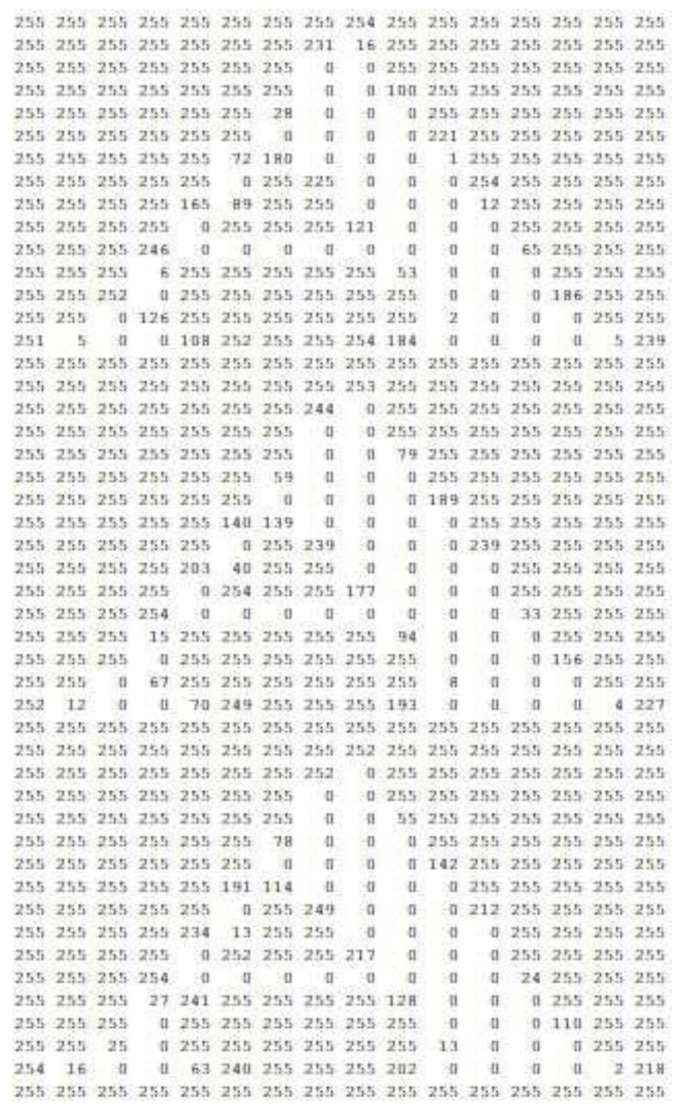
Extract from a.PATT file with an image pattern.

To use PATTERN type markers with this framework, they must be defined in PATT files. These files contain a mapping between the graphic content of the marker’s image and numeric values in the 0 to 255 range. Each value represents a color, in a gray scale, from 0 white to 255 black, as presented in Figure 22. An important indicator for the AR markers’ recognition is the confidence degree, which is a percentage number that defines the certainty of the recognition or the certain probability of a correct recognition.

To test the effects of the confidence degree in the AR marker recognition, we used PATTERN type markers (characters) with a 50% confidence degree, and it was verified that the system mismatched the “B” and the “F” markers. In another test, the confidence degree was lowered to 25% and the results were predictably worse. The “B” marker was now also mismatched with the “D” marker. In a third test, we used an 85% confidence degree, and all the markers (characters) were correctly recognized.

**Figure 23:**
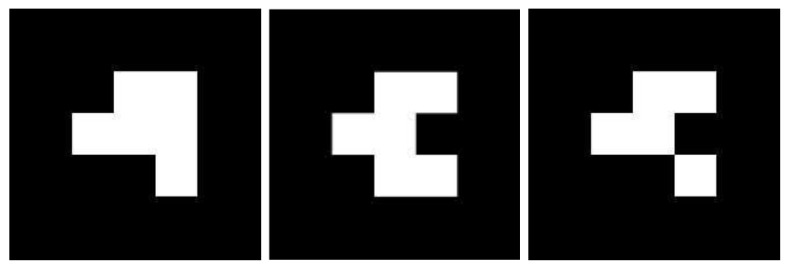
2D BARCODE markers.

When using 2D BARCODE type markers, visible in Figure 23, it is not necessary to include a definition file, which renders a much lighter processing. In the tests, like the ones previously described, the system always recognized the marker correctly with a confidence degree between 95% and 100%.

An additional conclusion is that the software library (the Threex.ARToolkit) had a software bug on the calculus of the confidence degree for 2D BARCODE type markers. It would always return a 100% confidence degree. The bug was corrected in the library and the calculation is now accurate.

**Figure 24:**
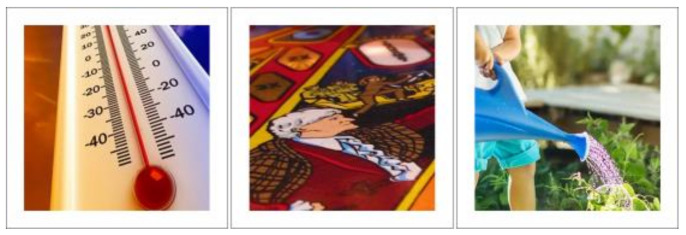
NFT markers.

Figure 24 illustrates an example of some Natural Feature Tracking (NFT) markers that can also be used with the ARToolkit framework. These types of markers allow for the usage of any image to create a customized marker, where it is also necessary to generate from the image for each tag a file with the unique keypoints to correctly identify the marker.

**Figure 25:**
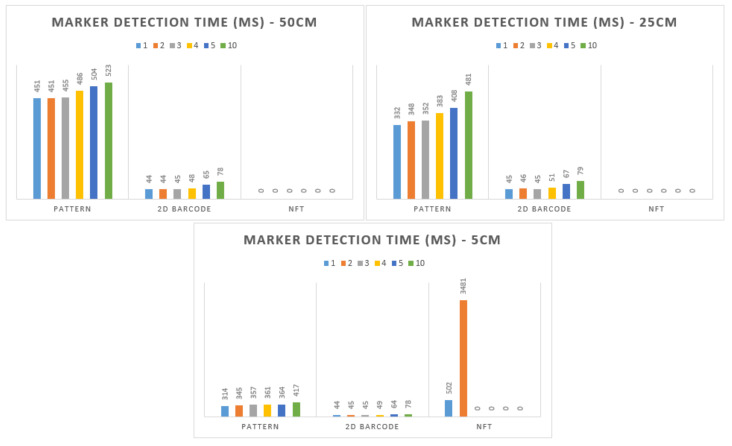
Marker detection time comparison (0 = not available).

The results of the tests carried out with the different types of markers are shown in Figure 25. These tests were executed 10 times each, with different distances (50, 25, and 5 cm) and different numbers of markers simultaneously (1, 2, 3, 4, 5, and 10). The values presented in the figure are the average values from the 10 iterations of each one.

The 2D BARCODE markers were the fastest to be detected by the system, while the NFT markers were the most time consuming and presented several problems in their detection, namely in terms of distance, where they were only detected at 5 cm from the camera and with more than two markers at the same time, where the system (on the smartphone) could not detect any AR marker.

For the AR markers to be used in the implementation, we chose the 2D BARCODE type as the detection time was lower, the recognition confidence was higher, the detection of multiple markers simultaneously was faster, and it did not need additional files to work.

### 5.2. AR Performance

**Figure 26:**
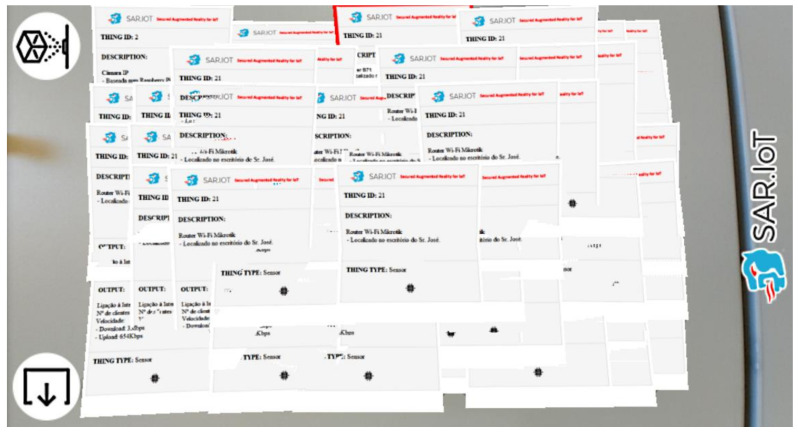
Performance test with multiple AR markers.

The solution exposed in this article was intended to work using a simple browser in a low-medium range smartphone, allowing the largest possible visualization of AR markers at the same tie, being the 60 markers simultaneously on the screen, the maximum allowed by the framework. In order to validate the capabilities of the system, it was necessary to verify the performance of the solution and whether it could handle a large number of markers captured by the system at the same time. For this purpose, several tests were carried out with the AR solution using 4 × 4 2D barcode markers (allowing a total of 8181 different markers), and in Figure 26, it is possible to see one of those tests, where 60 augmented information windows were generated at the same time on the same smartphone screen.

Overall, the system managed to always generate all the AR information windows needed, with only a slight drag in the animation when the number of AR markers recognized by the system was very high (as the picture above), but when zooming on a specific AR marker, this drag completely disappears.

### 5.3. Marker Security Protection

**Figure 27:**
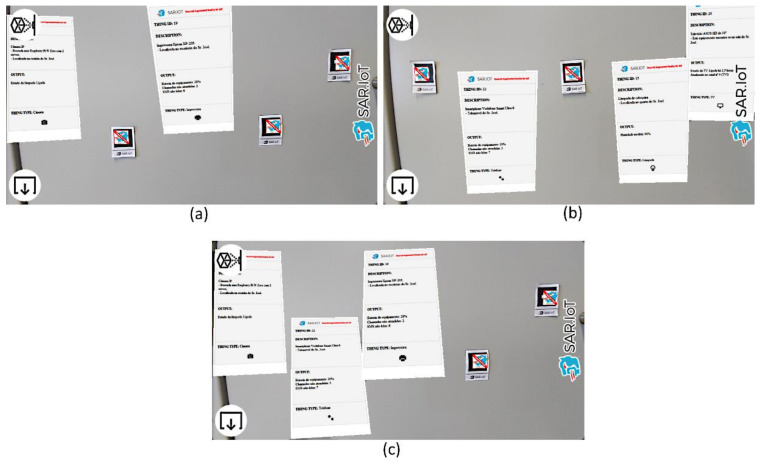
Available and not available AR markers for users U1 (**a**), U2 (**b**) and U3 (**c**).

To assure the security while retrieving the information from the IoT devices, several tests were conducted with a regular smartphone accessing the system, and although the system detects all the markers and processes them all, it only returns and displays the real-time information of the devices available to the current user, so every other device will have access denied and the AR marker will show that, as shown in Figure 27.

In the example above, we can see that the markers unlocked to users U1 (a) and U2 (b) were completely different, the two users had access to different devices, and the system only showed the information of the IoT devices that each user had permission for. Regarding user U3 (c), it could view and manage a set of devices to which users (a) and (b) also had access. This behavior is very useful, mainly in industrial or laboratorial situations, where different employees/users can only view or manage the IoT devices they have permissions for, which adds a security feature to who can view or not the information in real-time of each device using augmented reality.

## 6. Conclusions and Future Work

In this work, we proposed a real-time visualization system for IoT devices using consumer grade mobile phone devices and augmented reality. The principal objective was to be able to extract information in real-time from the IoT devices and present it using augmented reality without the need for additional specific hardware, keeping the solution low cost. The main contributions in this article were the creation of an architecture that allows the simplified use of augmented reality to visualize information in real-time from IoT devices with a security layer added to the AR, and the development of a functional prototype that demonstrates the operation of the proposed solution and validates the architecture.

The tests results concluded which type of AR marker was best to use and validated the security model used to protect the access to the information on the IoT devices.

In future work, an interesting approach to research would be the implementation of an AR marker generator, creating unique markers optimized for AR usage, mainly using a random quantity of triangles and rectangles, composing a unique pattern.

## Data Availability

Not applicable.
